# Nd:YAG Laser in the Treatment of Nail Psoriasis: Clinical and Dermoscopic Assessment

**DOI:** 10.5826/dpc.1102a140

**Published:** 2021-03-08

**Authors:** Yasmin Hesham Ali Elwan, Amira Abdel Azim, Michela Starace, Hala Shawky Abd Elhafiz

**Affiliations:** 1 Faculty of Medicine, Cairo University, Egypt; 2 Department of Dermatology and Venerology, Al Azhar University for Girls in Cairo, Egypt; 3 Department of Experimental, Diagnostic and Specialty Medicine-Division of Dermatology, University of Bologna, Italy

**Keywords:** Nd:YAG laser, nail psoriasis, NAPSI, dermoscopy

## Abstract

**Background:**

The Nd:YAG laser has emerged as a promising modality for the management of nail psoriasis owing to its ability for deep penetration of the skin surface, which has the advantage of destroying deep vessels.

**Objective:**

To assess the efficacy and safety of Nd:YAG laser in treating nail psoriasis.

**Methods:**

The present study was a randomized controlled study, conducted on 20 patients of both sexes (age older than 12 years) with mild to moderate psoriasis with nail involvement. We utilized facial telangiectasia parameters of Nd:YAG laser and beam diameter of 2.5 mm. Laser energy started with 110 J/cm^2^ in the first session and 130 J/cm^2^ in the rest of the sessions. Sessions were performed once monthly for up to 6 sessions.

**Results:**

We found no statistically significant difference in total Nail Psoriasis Severity Index (NAPSI) and nail bed scores before and after treatment among the treated group. However, there was statistically significant improvement in nail matrix score after treatment. On the other hand, the control group did not show any statistically significant changes for all scores throughout the study, except for the nail matrix score mean difference (0.35 ± 1.23 vs −1.00 ± 1.86 in the treated group). The degree of dermoscopic improvement was evident in the treated group (45% vs 25% in the control group). However, it was not statistically significant because of small sample size. The patients’ satisfaction and the external investigator’s assessment showed statistically significant negative correlation with total NAPSI mean difference in the treated group.

**Conclusion:**

The role of Nd:YAG laser in nail psoriasis is still controversial.

## Introduction

Nail psoriasis poses a therapeutic challenge to treating dermatologists; the severity and extension of nail involvement usually drives the treatment decision [1]. Laser therapy has shown to be effective and safe for nail psoriasis with high patient satisfaction. It could be used alone or combined with different therapeutic modalities [2]. The use of pulsed dye laser (PDL) to treat nail psoriasis has been explored. The best response was observed in onycholysis and subungual hyperkeratosis [3]. PDL in combination with topical tazarotene showed significantly better improvement compared to tazarotene monotherapy [4]. Excimer laser has been approved for the treatment of psoriasis since 2000. However, so far in the available literature, excimer laser has not been found to be effective in a limited number of patients of nail psoriasis. This could be because of the poor penetration of UVB in the human nail plate. Results of excimer laser treatment of nail psoriasis are poor and time-consuming compared to PDL [5]. Recent reports demonstrated that the Nd:YAG laser exhibited promising effectiveness and a well-tolerated safety profile in the management of nail psoriasis [6]. In the setting of nail psoriasis, dermoscopy was reported to be an effective tool for early assessment of nail lesions, as well as in differentiating the psoriatic lesions from other disorders [7]. Moreover, dermoscopy can be used for evaluation of response to treatment for nail psoriasis. Thus, we conducted the present randomized controlled study to assess the efficacy and safety of Nd:YAG laser in treating nail psoriasis, based on clinical and dermoscopic assessment.

## Patients and Methods

The initiation and patient enrollment of the study preceded the official approval of the local ethics committee of the participating institution.

### Study Design and Patients

The present study was a randomized, within-patient, controlled study that was conducted on 20 patients who were recruited from the Dermatology and Venereology Outpatient Clinic of AL-Zahraa University Hospital during the period between January 2019 and March 2020. Both sexes were included if they were older than 12 years old and had mild to moderate psoriasis associated with nail involvement. The diagnosis was based upon clinical characteristics of psoriasis (erythematous papules and plaques covered by silvery white scales with psoriatic nail changes). Patients were instructed to stop any systemic or topical treatments for the nails 2 months before the study. We excluded patients who refused to sign the informed consent, patients who were eligible for systemic therapy, patients with pustular or erythrodermic psoriasis, patients with severe psoriatic arthritis needing systemic treatment, patients with onychomycosis, and patients who received systemic anti-psoriatic treatment 2 months prior to study’s enrollment.

### Sampling and Randomization

We utilized a probability, simple, random sampling technique for patient recruitment. In each eligible patient, a coin toss was used to treat a randomly allocated finger or toenail of one side, while the other side was left untreated.

### Study Intervention

The eligible patients were treated with laser sessions by Synchro FT Nd: YAG laser (DEKA Laser). We used the facial telangiectasia parameters of Nd: YAG laser of a beam diameter of 2.5 mm and started laser energy with 110 J/cm^2^ in the first session and 130 J/cm^2^ in the rest of the sessions, single pulse frequency and shallow depth. All patients were advised before every session to apply topical anesthetic cream (containing lidocaine and prilocaine) to avoid pain during laser sessions. Sessions were performed once monthly for up to 6 sessions.

### Evaluation and Follow-up

A full history was taken of all eligible patients along with, dermatological examination for psoriatic lesions, photography of the nails (Galaxy A30 phone camera; dual m16 MP, f/1.7, 27 mm [wide], PDAF; 5 MP, f/2.2, 12 mm, [ultra wide], and a clinical assessment by the Nail Psoriasis Severity Index (NAPSI). The evaluation was done at baseline and was repeated 1 month after the end of treatment.

The subjective evaluation involved both patients and investigators. Every patient was asked about his/her self-satisfaction of the results of the treatment after the last session using a visual analog scale (VAS); a rating of 0 for no satisfaction and a rating of 10 for the best satisfaction. The external investigator expressed the degree of improvement in percentages. Using percentile and quartile ranges, patients were evaluated for degree of improvement as follows: mild improvement ≤ 25%, moderate improvement, 26–50%, marked improvement, 51–75%, excellent improvement, 76–100%.

In addition, a clinical assessment by the NAPSI score was done for each patient. The NAPSI is a numerical score for scaling the severity of psoriatic lesions of the nail. The score assesses the involved areas within each nail covering the nail bed (score range 0–4) and nail matrix (score range 0–4) lesions. A composite score for each nail is then calculated (range 0–8). The final score is the sum of all nails score that ranges 0–160 [8].

The dermoscopic evaluation was done using a DermLite HUD Dermatoscope (polarized light, magnifying lens ×10, connected to the mobile phone camera magnifying up to ×4×). The examination was done by a visual expert opinion method (by Dr. Michela Starace). The expert looked at the typical dermoscopic signs of nail psoriasis. These signs were checked if they were present or not in the treated and in the control nails 1 month after the last session.

### Statistical Analysis

The data were processed and analyzed using a statistical package for social sciences (SPSS, version 20.0 Chicago, Illinois, USA). The mean (± SD [standard deviation]) or median (range) were used to present the continuous data. The percentages were used to quantify the qualitative data. The association analysis was done by paired t test and chi-square test for quantitative and qualitative data. Spearman correlation test was used to examine the correlation between quantitate variables. A P value < 0.05 was significant.

## Results

A total of 20 patients were included in the present study. The mean age of the included patients was 40.90 ± 17.02 and 65% of them were females. Regarding the duration of psoriasis (in years) among the studied groups, the median was 9 and half of the group were between 6–13.5; and the median duration of nail psoriasis (in years) among the studied groups was 3 and half of the group were between 2–9. One-fourth of the patients were diabetic and 10.0% were hypertensive. In each patient the number of affected nails in the treated hand or foot was 5 (Table 1).

**Figure 1:**
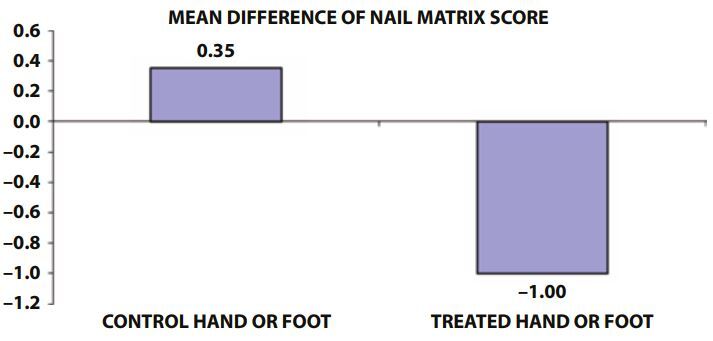
Mean difference of nail matrix score between the control and the treated groups.

In the Nd:YAG-treated side, there were no statistically significant changes in total NAPSI and nail bed score after the end of the study (P > 0.05). On the other hand, there was a significant decrease in nail matrix score after treatment (19.55 ± 1.15 versus 18.55 ± 2.78; P = 0.027). In the control group, there were no statistically significant changes in any of total NAPSI scores after the end of the study (P > 0.05). The treated group had a significantly higher reduction in the nail matrix score than the control group at the end of treatment (1.00 ± 1.86 versus 0.35 ± 1.23, respectively; P = 0.01; Table 2) (Figure 1).

**Figure 2:**
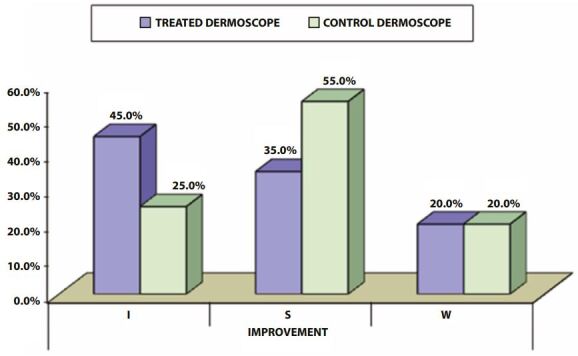
The degree of dermoscopic improvement in the treated and control groups.

Regarding the dermoscopic assessment in the treated group, 9 cases (45.0%) showed improvement, 7 cases (35%) showed stable disease, and 4 cases (20.0%) showed worsening of disease. In the control group 5 cases (25.0%) improved, 11 cases (55.0%) remained stable, and 4 cases (20.0%) worsened with no statistically significant difference between both groups (Figure 2). The number of dermoscopically improved cases was higher in the treated group [6 (30%), 2 (10%), 1 (5.0%), 3 (15.0%), and 2 (10.0%)] than the control group [4 (20.0%), 0 (0.0%), 0 (0.0%), 1 (5.0%) and 1 (5.0%)] in onycholysis with erythematous border, pitting, salmon patches, subungual hyperkeratosis, and trachyonychia, respectively. There were no cases of improvement in crumbling in both groups. No statistically significant differences between both groups were detected in any of the above lesions (Table 2).

Regarding the degree of improvement as described by the patients, the median was 5, with half the patients statistically improved from 2 to 8. Regarding the degree of improvement, as described by the external investigator, the median (by percentage) was 27.5%, with half the patients statistically improved from 12.5% to 55% (P < 0.05; Table 3). The only side effect encountered in our study was mild pain in (30.0%) of the patients. The rest of the patients (70.0%) did not have any side effects.

## Discussion

The results of the present study demonstrated that the Nd;YAG laser only improved the nail matrix lesions in our patients, with no significant improvement in the nail bed lesions or total clinical score. Dermoscopically, the Nd;YAG laser led to notable improvement in the nail lesions; however, this improvement did not reach the level of statistical significance. The patient satisfaction and investigator’s opinion were significantly favorable after the end of treatment.

The development of an objective tool for clinical response to treatment is one of the main challenges during the management of nail psoriasis [9]. Since its validation by Rich and Scher in 2003 [8], the NAPSI has shown to be a valid tool for clinical evaluation of the degree of nail involvement in psoriasis, as well as the nail response to treatment.

In the present study we found that the Nd:YAG laser led to statistically insignificant decrease in total NAPSI and nail bed scores after the end of treatment. On the other hand, there was a significant decrease in nail matrix score after treatment. Such findings were contrary to the study by Khashaba et al. [10] and another study by Kartal et al. [6] that found significant reduction in NAPSI among nail psoriasis patients after Nd: YAG laser treatment.

**Figure 3:**
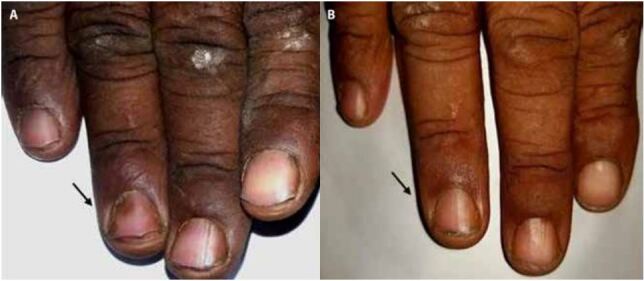
Clinical picture of a 62-year-old male patient with (A) fingernail psoriasis showing salmon patches before treatment and (B) improvement after 6 treatment sessions.

**Figure 4:**
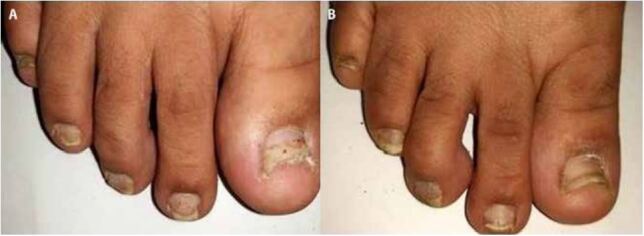
Clinical picture of a 27-year-old female patient with toenail psoriasis showing (A) onycholysis and subungual hyperkeratosis before treatment that (B) improved after 6 treatment sessions.

The exact causes of heterogeneity between our findings and the above-mentioned studies are unclear; however, such heterogeneity can be explained by many factors. First, the difference in the site of the treated nails between our study and the above-mentioned reports might have contributed to this heterogeneity. Moreover, in the above studies, they treated fingernails only, while we treated finger and toenails; toenails may be more resistant to treatment than fingernails. Second, different parameters of laser applications can represent another explanation for this heterogeneity in clinical response to Nd:YAG. In our study, we used the facial telangiectasia parameters of Nd: YAG laser by a beam diameter of 2.5 mm. Laser energy started with 110 J/cm^2^ in the first session and 130 J/cm^2^ in the rest of the sessions, single pulse frequency, and shallow depth for up to 6 sessions. Khashaba and colleagues [10] utilized a 5 mm spot size and 40 J/cm^2^ fluence, in partially overlapping mode in each session for 4 sessions. Kartal and colleagues [6] utilized a beam diameter 6 mm, laser energy was 10 J/cm^2^ with 1.5 Hz repetition rate for 3 sessions Third, the limitations of the NAPSI score itself may represent another factor. Previous reports demonstrated that NAPSI has poor correlation with the clinical severity of nail psoriasis [11]. In addition, the method of lesion assessment by NAPSI is a limitation as well; the assessment depends solely on the presence of a lesion (bed or matrix) within each quadrant, regardless of the presence of other lesions or the severity of this lesion. Last, our small sample size may represent another factor explaining the insignificant findings. The speculation of the impact of small sample size in our study is supported by the significant improvement in patient satisfaction and investigator’s opinion. Thus, we can hypothesize that the improvement in nail features was notable; however, it did not reach the level of statistical significance because of small sample size (Figures 3 and 4).

## Conclusions

**Figure 5:**
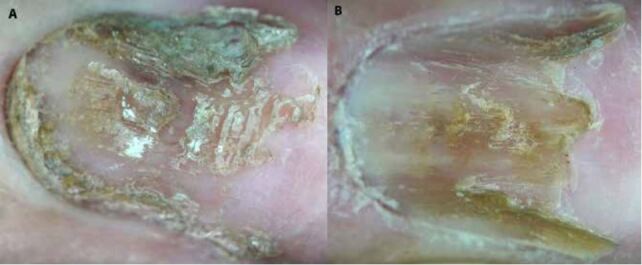
Dermoscopic picture of a 60-year-old male patient with fingernail psoriasis showing (A) onycholysis before treatment that (B) improved after 6 treatment sessions.

**Figure 6:**
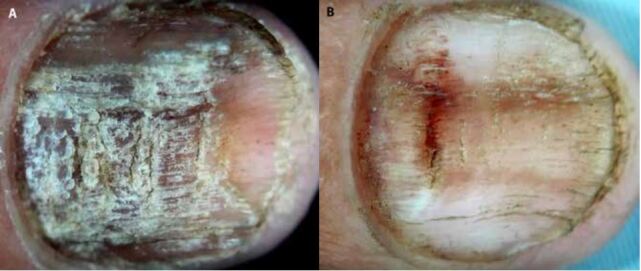
Dermoscopic picture of 19-year-old female patient with fingernail psoriasis showing (A) trachyonychia before treatmentthat (B) improved after 6 treatment sessions.

Nail psoriasis may be present with an extremely wide spectrum of symptoms, which vary in severity and type. Dermoscopy demonstrates high efficacy in common, as well as rare, features of nail involvement [12]. In the present study, we assessed the effect of Nd:YAG laser on the improvement of dermoscopic features of the nails. Our results demonstrated no statistically significant difference between the control and the treated groups regarding the percentage of improved lesions. This finding was consistent regardless of the type of lesion. These results contradict the previous findings by Khashaba et al [10]. This may be because the dermoscopic assessment in the present study relied on the visual expert opinion method, which is a subjective method with a high chance of low inter-rater reliability. The small sample size may represent another factor explaining the insignificant findings of our results. The degree of improvement was evident in the treated group (45% versus 25% in the control group). However, the small sample size might have hindered the effect size from reaching the margin of statistical significance (Figures 5 and 6).

Side effects of Nd:YAG laser treatment are usually minor and may include pain during treatment, redness, swelling and itching immediately after the procedure that may last for few days [13]. In the present study, 6 patients (30.0%) suffered from mild pain and 14 patients (70.0%) had no side effects during laser sessions.

The present study is one of the few reports that assess the efficacy and safety of Nd:YAG laser in treating nail psoriasis both clinically and dermoscopically. The advantages of our study include random allocation of the patients, ensuring low selection bias; the presence of a control group ensuring low performance bias; and the use of both clinical score and dermoscopic features. However, we acknowledge that the present study has some limitations. The study was a single-center experience and therefore the results cannot be generalized to the general population. The sample size of the present study was relatively small and might have hindered the outcome from reaching the margin of statistical significance. The use of a subjective method for dermoscopic assessment is another limitation.

In conclusion, Nd:YAG laser significantly improves the satisfaction of the patients with nail psoriasis, with minimal side effects. However, its role on clinical severity score and dermoscopic features is still controversial.

## Figures and Tables

**Table 1:** Demographic and Clinical Characteristics of the Included Patients

		Total no. = 20

Age	Mean ± SD	40.90 ± 17.02

	Range	17–64

Sex	Female	13 (65.0%)
	Male	7 (35.0%)

Occupation	Student	4 (20.0%)
	Housewife	7 (35.0%)
	Farmer	3 (15.0%)
	Painter	2 (10.0%)
	Employee	2 (10.0%)
	Ex-employee	1 (5.0%)
	Worker	1 (5.0%)

Skin phototype	II	1 (5.0%)
	III	7 (35.0%)
	IV	11 (55.0%)
	V	1 (5.0%)

Duration of psoriasis (years)	Median (IQR)	9 (6–13.5)

	Range	2–22

Duration of nail psoriasis (years)	Median (IQR)	3 (2–9)

	Range	1–14

Associated diseases	None	13 (65.0%)
	Diabetic	5 (25.0%)
	Hypertensive	2 (10.0%)

Treated hand or foot	Hand	10 (50.0%)
	Foot	10 (50.0%)

Number of affected nails	5	20 (100.0%)

IQR = interquartile range.

**Table 2:** NAPSI Scores and Dermoscopic Assessment Among the Treated Group Before Start vs After End of Study

Before		Nd:YAG Group			Control Group			P value
		After	P value	Before	After	P value		
Total NAPSI	Mean ± SD	33.90 ± 2.67	32.60 ± 4.45	0.113	32.15 ± 4.22	32.55 ± 4.43	0.46	0.082
	Range	30–40	23–40		17–36	19–37		
Nail bed score	Mean ± SD	14.35 ± 2.70	14.05 ± 3.30	0.632	13.40 ± 2.44	13.45 ± 3.03	0.92	0.66
	Range	10–20	10–20		10–17	7–17		
Nail matrix score	Mean ± SD	19.55 ± 1.15	18.55 ± 2.78	0.027	18.75 ± 4.44	19.10 ± 3.34	0.21	0.01
	Range	15–20	9–20		0–20	5–20		
Improvement by dermoscopy	I	9 (45.0%)			5 (25.0%)			0.32
	S	7 (35.0%)			11 (55.0%)			
	W	4 (20.0%)			4 (20.0%)			
Onycholysis with erythematous border	I	6 (30.0%)			4 (20.0%)			0.753
	S	11 (55.0%)			13 (65.0%)			
	W	3 (15.0%)			3 (15.0%)			
Pitting	I	2 (10.0%)			0 (0.0%)			0.146
	S	18 (90.0%)			20(100.0%)			
	W	0 (0.0%)			0 (0.0%)			
Crumbling	I	0 (0.0%)			0 (0.0%)			0.311
	S	19 (95.0%)			20(100.0%)			
	W	1 (5.0%)			0 (0.0%)			
Salmon patches	I	1 (5.0%)			0 (0.0%)			0.598
	S	18 (90.0%)			19 (95.0%)			
	W	1 (5.0%)			1 (5.0%)			
Subungual hyperkeratosis	I	3 (15.0%)			1 (5.0%)			0.505
	S	16 (80.0%)			17 (85.0%)			
	W	1 (5.0%)			2 (10.0%)			
Trachyonychia	I	2 (10.0%)			1 (5.0%)			0.513
	S	18 (90.0%)			18 (90.0%)			
	W	0 (0.0%)			1 (5.0%)			

I = improved; NAPSI = nail psoriasis severity index; S = stable; SD = standard deviation; W = worsened.

**Table 3:** Degree of Improvement by the Patient (Patient Satisfaction) and the External Investigator

		Total n = 20
Degree of improvement by patient (patient satisfaction) (%)	Median (IQR)	5 (2–8)
	Range	0–9
Degree of improvement by the external investigator (%)	Median (IQR)	27.5 (12.5–55)
	Range	0–80

IQR = interquartile range.

## References

[1] Bardazzi F, Starace M, Bruni F, Magnano M, Piraccini BM, Alessandrini A (2019). Nail psoriasis: an updated review and expert opinion on available treatments, including biologics. Acta Derm Venereol.

[2] Arango-Duque LC, Roncero-Riesco M, Bárcena TU, Álvarez IP, López EF (2017). Treatment of nail psoriasis with pulse dye laser plus calcipotriol betametasona gel vs. Nd:YAG plus calcipotriol betamethasone gel: an intrapatient left-to-right controlled study. Actas Dermosifiliogr.

[3] Yin N, Choudhary S, Nouri K (2013). Pulsed dye laser for the treatment of nail psoriasis. Cutis.

[4] Huang YC, Chou CL, Chiang YY (2013). Efficacy of pulsed dye laser plus topical tazarotene versus topical tazarotene alone in psoriatic nail disease: a single–blind, intrapatient left–to–right controlled study. Laser Surg Med.

[5] Al-Mutairi N, Noor T, Al-Haddad A (2014). Single blinded left-to-right comparison study of excimer laser versus pulsed dye laser for the treatment of nail psoriasis. Dermatol Ther.

[6] Kartal SP, Canpolat F, Gonul M, Ergin C, Gencturk Z (2018). Long-pulsed Nd:YAG laser treatment for nail psoriasis. Dermatol Surg.

[7] Yadav TA, Khopkar US (2015). Dermoscopy to detect signs of subclinical nail involvement in chronic plaque psoriasis: A study of 68 patients. Indian J Dermatol.

[8] Rich P, Scher RK (2003). Nail Psoriasis Severity Index: A useful tool for evaluation of nail psoriasis. J Am Acad Dermatol.

[9] Zalaudek I, Argenziano G, Di Stefani A (2006). Dermoscopy in general dermatology. Dermatol.

[10] Khashaba SA, Gamil H, Salah R, Salah E (2019). Efficacy of long-pulsed Nd-YAG laser in the treatment of nail psoriasis: a clinical and dermoscopic evaluation. J Dermatol Treat.

[11] Klaassen KMG, Van De Kerkhof PCM, Bastiaens MT, Plusjé LGJM, Baran RL, Pasch MC (2014). Scoring nail psoriasis. J Am Acad Dermatol.

[12] Yadav TA, Khopkar US (2015). Dermoscopy to detect signs of subclinical nail involvement in chronic plaque psoriasis: A study of 68 patients. Indian J Dermatol.

[13] Shen JH, Chang CC, Chen YT, Hsih CJ, Huang H, Lin BS (2016). Using a low fluence Q-switched 532/1064-nm Nd:YAG laser for facial skin depigmentation in asian patients: outcome and complication profile analysis. Ann Plast Surg.

